# The application of intraoperative ankle dislocation approach in the treatment of the unstable trimalleolar fractures involving posterior ankle comminuted fracture: a retrospective cohort study

**DOI:** 10.1186/s12893-018-0356-9

**Published:** 2018-04-18

**Authors:** Wenzhao Xing, Peng Xie, Linjie Wang, Changcheng Liu, Jian Cui, Zhiguo Zhang, Liang Sun

**Affiliations:** 1 Department of Orthopaedics, the Third Hospital of Hebei Medical University, No.139, Ziqiang Road, Shijiazhuang, 050000 People’s Republic of China; 2 Department of Nuclear Medicine, the Third Hospital of Hebei Medical University, Shijiazhuang, 050000 China; 3 Department of Orthopaedics, Affiliated Hospital of Hebei University, Baoding, 071000 People’s Republic of China

**Keywords:** Trimalleolar fractures, Posterior ankle comminuted fracture, Intraoperative dislocating ankle joint method

## Abstract

**Background:**

The aim of this study was to introduce a novel intraoperative lateral ankle dislocation approach during surgical treatment for patients with unstable trimalleolar fractures involving posterior ankle comminuted fractures and compare its effects and safety with those with conventional approach.

**Methods:**

From June 2006 to June 2014, 69 patients diagnosed as unstable trimalleolar fractures involving posterior ankle comminuted fracture were included in this study. The patients were divided into intraoperative dislocating ankle group (experimental group) and conventional treatment group (control group) according to surgical modalities. The following parameters including rate of primary healing, healing time, incidence of talus necrosis, incidence of post-traumatic arthritis, functional outcomes according to Baird-Jackson classification system, and any possible complications in two groups were recorded and compared.

**Results:**

There were no significant differences regarding the rate of primary healing, healing time and the rate of talus necrosis in two groups (*P* > 0.05). The incidence of post-traumatic arthritis in experimental and control group were 0 and 24.24% (*P* = 0.0006), respectively. The rate of excellent and good outcomes were achieved in 91.67% in experimental group and 72.73% in control group (*P* = 0.038), respectively.

**Conclusions:**

The findings suggest that the intraoperative ankle dislocation approach appears to be a promising surgical option for unstable trimalleolar fractures involving posterior ankle comminuted fracture because it can provide better functional outcomes and lower incidence of post-traumatic arthritis while not compromising primary healing and healing time.

## Background

Ankle fracture is an injury to bones of the ankle joint, with an annual incidence of 174 out of every 100,000 people [1]. It represents about one-tenth of all fractures [2]. With inappropriate treatment or even appropriate treatment, 10% of patients with ankle fractures develop complications such as post-traumatic arthritis, limited ankle activity, and chronic ankle pain. In severe cases, ankle arthrodesis may be required which will lead to loss of joint function or even impairs the daily activities of the patients [3–5].

Ankle fractures can be classified based on the number of malleoli involved, namely unimalleolar, bimalleolar or trimalleolar fractures [6]. Among these fractures, trimalleolar fracture is the most severe type. This fracture involves the medial and lateral malleoli and the anterior or posterior margin of the tibia. The fracture is usually caused by a strong external rotation force which will severely damage ankle-stabilizing structures such as the bones and ligaments. Generally, stable fractures are treated with cast immobilisation, whereas unstable fractures are mainly treated by internal fixation [7–9]. Unstable ankle fractures are usually associated with fracture dislocations and displacement of the malleoli. Therefore, such unstable fractures usually require early reduction and surgical management to enable stabilization and early mobilization. This is because inadequate reduction often results in early osteoarthritis, pain and loss of ankle joint function [10–12]. However, it is very difficult to achieve fracture reduction in cases associated with comminuted posterior malleolar fractures involving the articular cartilage. Furthermore, if there exist malreduction, patients may develop post-traumatic arthritis of the ankle, which would severely affect their prognosis and quality of life. Currently, the reduction and fixation of the associated medial and lateral malleoli have been well established due to the superficial location of these structures. However, the exposure and fixation of the posterior malleolar fracture remain controversial due to its deep position and proximity to peripheral nerves, blood vessels, and tendons.

The conventional surgical approach to the posterior malleolar fracture includes medial or posteromedial approach and posterolateral surgical approach alone, and/or in combination [13–17]. Based on the pathological classification of posterior malleolar fractures proposed by Haraguchi et al. [18], the posterolateral approach is more suitable for type I fractures (posterolateral-oblique fractures), while the conventional medial approach or posteromedial approach is more advantageous for type II fractures (medial-extension fractures). However, none of the above approaches can expose the articular surface sufficiently for type III fractures (comminuted fractures, i.e., complicated posterior malleolar fractures). Currently, the optimal surgical approach for the fixation of comminuted posterior malleolar fractures remains controversial [18, 19]. To date, little effort has been done to investigate the treatment approaches for trimalleolar fractures involving posterior comminuted fractures, and there is no definite method and criteria for trimalleolar fractures involving posterior comminuted fractures. The aim of this study was to introduce a novel intraoperative lateral ankle dislocation approach during surgical treatment and compare its effects and safety with those with conventional approach for patients with unstable trimalleolar fractures involving comminuted posterior malleolar fractures.

## Methods

### Patients

This study was approved by the ethics committee of the Third Hospital of Hebei Medical University and conducted in accordance with the Declaration of Helsinki. All subjects signed consent forms. We retrospectively collected the clinical data of patients with unstable trimalleolar fractures involving comminuted posterior malleolar fractures who were treated at the Third Hospital of Hebei Medical University from June 2006 to June 2014. The diagnosis of unstable trimalleolar fracture is based on spontaneous dislocation of ankle joint on physical examination and ankle joint dislocation or subluxation, separation of the distal parts of the fibula and tibia on radiographic examination. Preoperatively, all patients underwent physical examinations and posteroanterior and lateral X-ray and CT examinations of the ankle X-ray and CT examinations to determine the diagnosis and type of ankle fracture.

Inclusion criteria were as follows: (1) trimalleolar fracture, (2) complete separation of the distal parts of the fibula and tibia, (3) posterior malleolar fracture involving more than 25% of the distal articular surface of the tibia or articular gapping of more than 2 mm, and (4) comminuted posterior malleolar fracture (i.e., small comminuted fractured fragments located between the displaced posterior malleolus fracture fragments and the front side of the main distal tibial fracture fragment).

Exclusion criteria were as follows: (1) open fracture, (2) injuries of the surrounding blood vessels and nerves, (3) stable trimalleolar fracture, (4) contraindication to surgery due to severe systematic disease, (5) old fracture for more than 4 weeks, and (6) poor compliance.

### Surgical procedures

#### Preoperative routine treatment

**Fig. 1:**
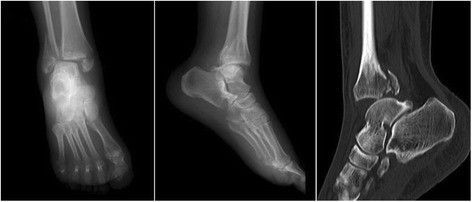
Preoperative X-ray and CT images showed unstable trimalleolar fractures involving posterior ankle comminuted fracture

After admission, patients were required to lift the injured limb and take medications to alleviate the swelling. Posteroanterior and lateral X-ray and CT examinations of the ankle were performed to determine the diagnosis and type of ankle fracture (Fig. 1). A surgical plan was formulated, and any comorbidity was treated as well. Surgery was performed once the soft-tissue swelling had been alleviated, provided no contraindication to surgery. Antibiotics were administered both pre- and postoperatively to prevent wound infection. Antibiotic treatment is essential since it has been reported that the rate of wound infection in patients with ankle fractures can reach up to 10% [20, 21].

#### Intraoperative ankle dislocation approach

**Fig. 2:**
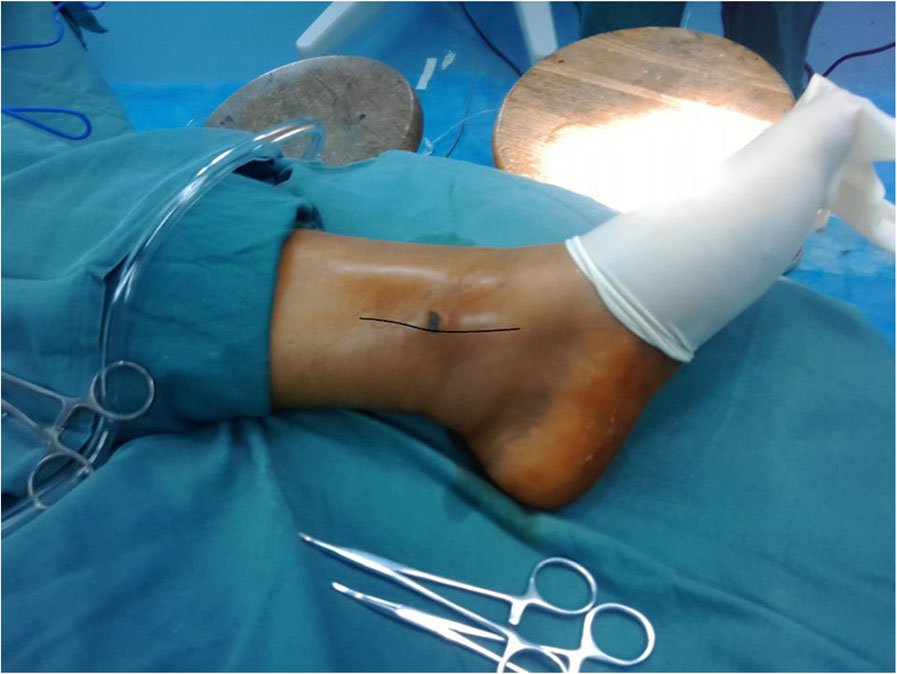
An 8-cm longitudinal incision

**Fig. 3:**
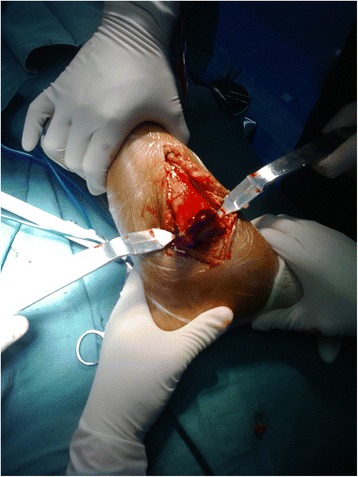
The distal articular surface of the tibia was exposure after dislocation

**Fig. 4:**
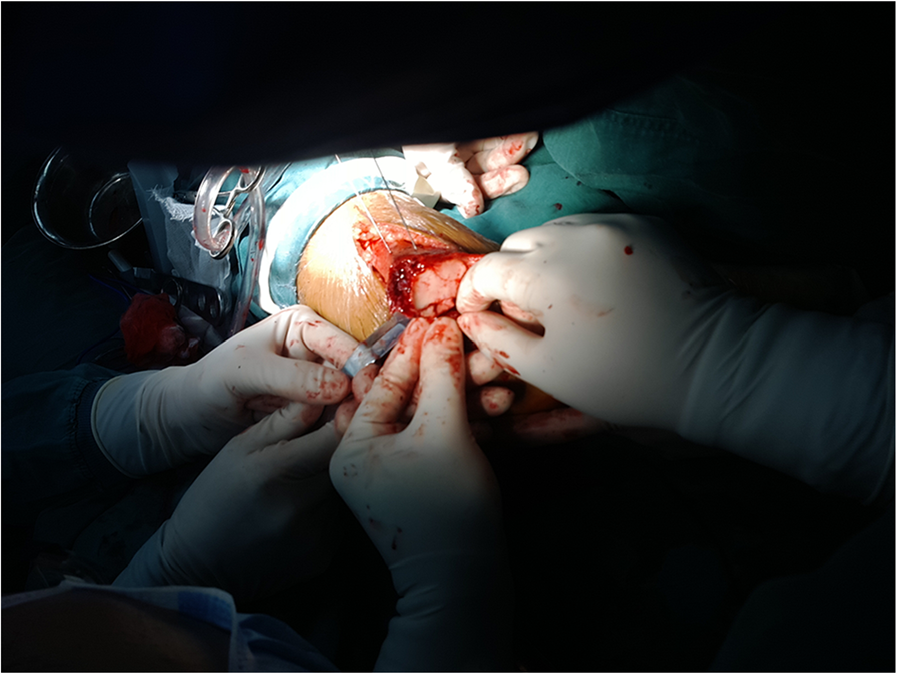
The posterior malleolar fracture after separation was anatomically reduced to the distal ends of the tibia under direct vision and then internally fixed with Kirschner wire

**Fig. 5:**
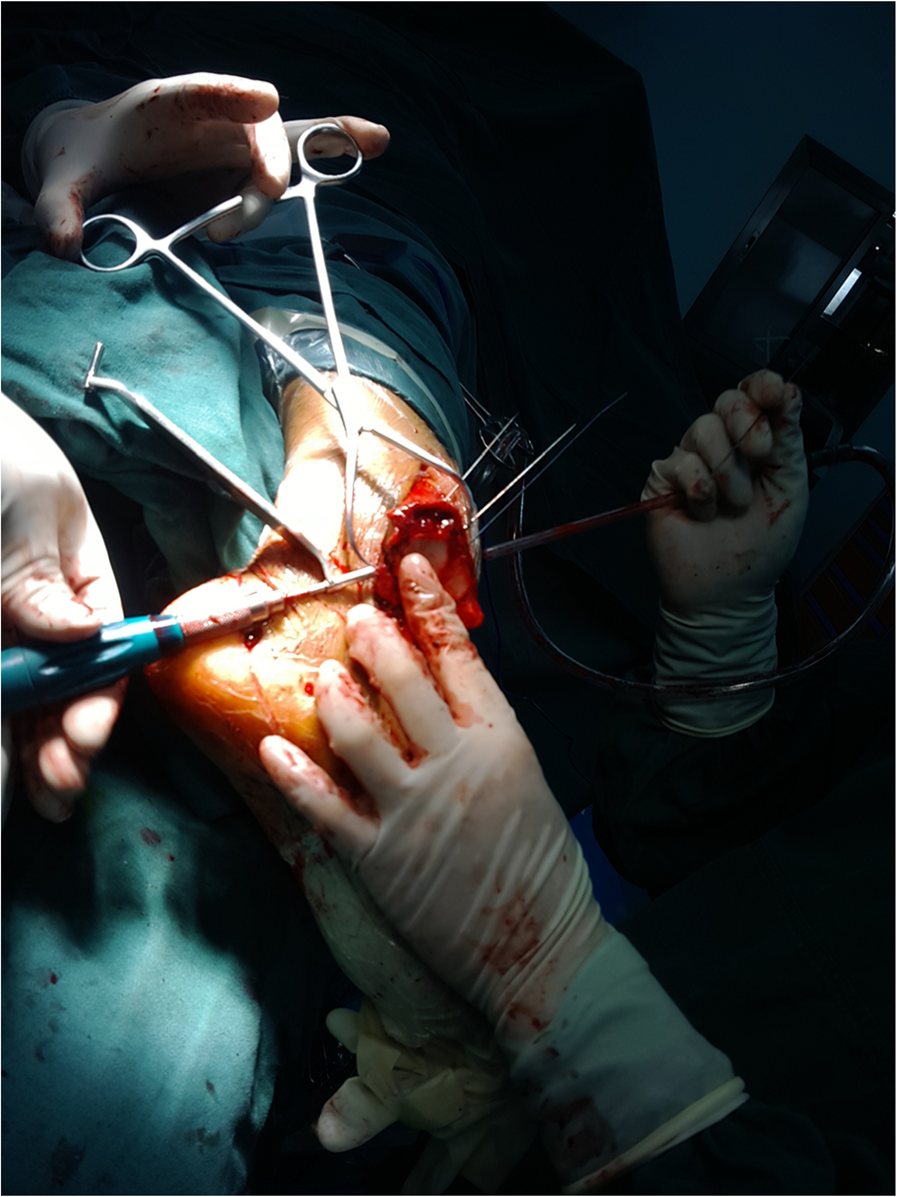
The posterior malleolus fractures were internally fixed with absorbable cartilage nails combined with Kirschner wire

**Fig. 6:**
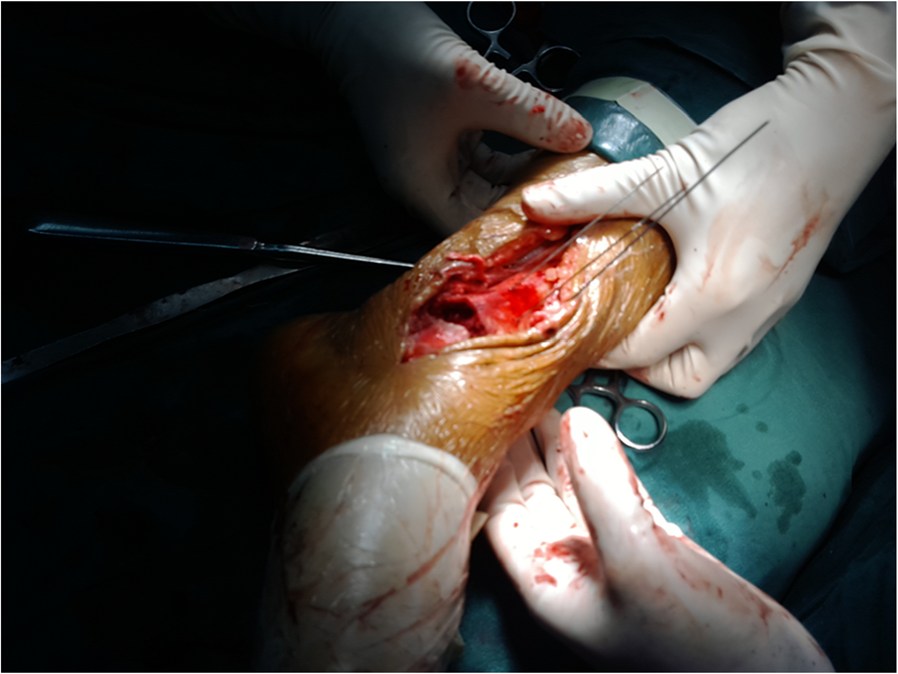
The previously dislocated ankle joint was reduced

**Fig. 7:**
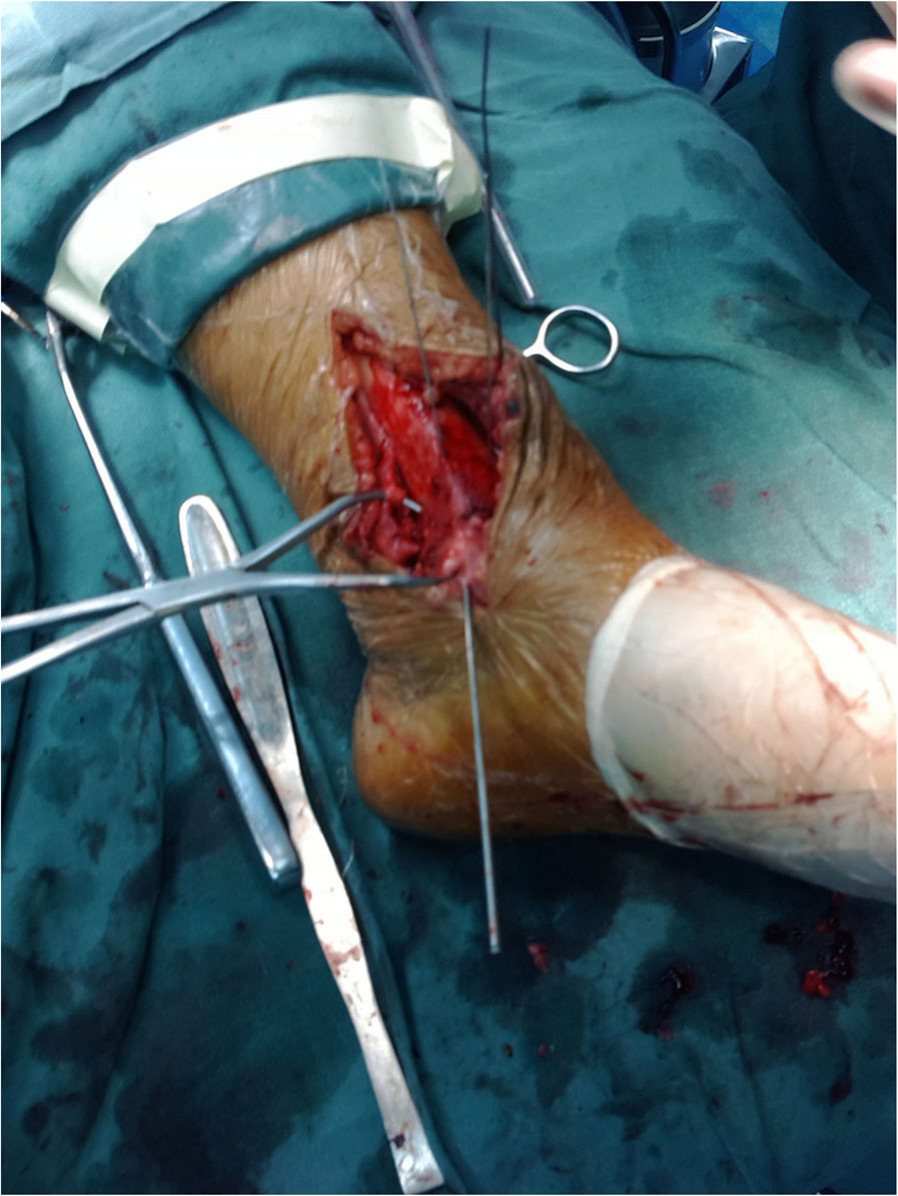
The reduction and fixation of medial malleolus fracture

**Fig. 8:**
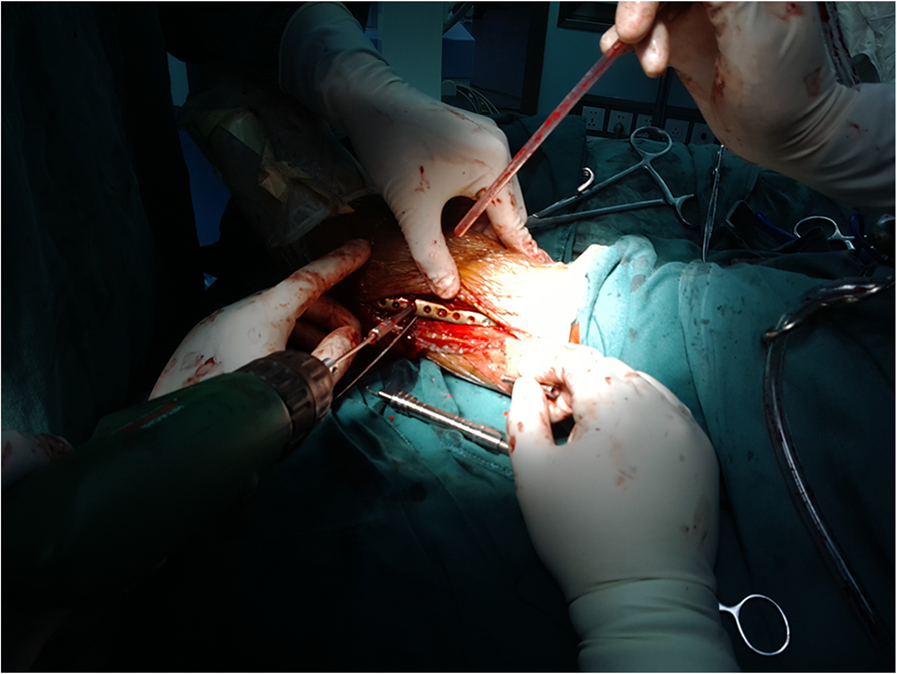
The reduction and fixation of lateral malleolus fracture by a steel plate

In the experimental group, surgery was performed using pneumatic tourniquets with the patient lying in a supine position [22]. After routine disinfection, an 8-cm skin incision was made along the middle line of the medial side of the tibia towards the tip of the medial malleolus (Fig. 2). The fracture fragments of the medial malleolus were exposed. Attention should be paid to protect the great saphenous vein during the dissection. A periosteal detacher which was tightly attached to the tibial surface was used to perform blunt dissection until the posterior malleolus. The fracture fragments of the medial malleolus were lifted distally. Then the talus and distal fracture stumps of the medial and lateral malleoli were pulled laterally to achieve lateral dislocation of the ankle (Fig. 3). During the process of dislocation, direct or indirect excessive force is not allowed to perform dislocation because it may increase the risk of uncontrollable injuries. If there exists difficulties in dislocations, the surgeon should re-examine the fracture type, incision length and the range of soft tissue peeling. Soft tissue peeling should not be reduced for pursuing minimal invasiveness. In addition, soft tissue peeling should be as little as possible provided that the dislocation was not affected. Thus, the posterior malleolus and articular surface of the distal tibia were fully exposed. Small fractured cartilage pieces were searched from the fracture fragments of the posterior malleolus, and attached to the main part of the posterior malleolar fracture with a medical aural and encephalic (EC) glue (catalog number: 050419; Guangzhou Baiyun Medical Adhesive Co. Ltd., Guangzhou, P. R. China). Then, the posterior malleolar fracture was anatomically reduced and internally fixed with absorbable screw, metallic screw, and/or Kirschner wire (Figs. 4 and 5). Next, the previously dislocated ankle joint was reduced (Fig. 6), followed by reduction of the medial malleolar fracture fragments with cannulated screw, metallic screws and/or Kirschner wire (Fig. 7). For the lateral malleolus, a lateral incision was made on the fibula to expose the fracture stump, and a plate was applied to achieve fracture reduction (Fig. 8). The wound was washed with normal saline solutions (0.9%) and the incision was closed using interrupted sutures. The ankle joint was immobilized in a gypsum plaster cast for 4–6 weeks. After removing the gypsum, the situation of fracture healing was evaluated by X-ray and CT examination if necessary. If the state of fracture healing was satisfactory, the dorsiflexion and plantar flexion functional exercise without loading could be carried out. After surgery, affected limb elevated exercise was routinely conducted to decrease the swelling. In addition, mannitol infusions (250 ml) were administered twice daily for 3 days and discontinued unless there was no obvious wound swelling. Two to three months after surgery, squatting could be added gradually to strengthen flexion and extension degree of the ankle joint. Three to six months after surgery, full weight-bearing activities could be performed after the fracture union.

#### Conventional surgical approach

In the control group, the posterolateral surgical approach was used in 22 patients, while the medial approach was used in 11 patients. In the case of the posterolateral surgical approach, an incision was made along the posterior margin of the fibula to expose the posterior malleolar facture. The fracture reduction was performed in leverage and fixed using a Kirschner wire inserted from the anterior to posterior direction or from the posterior to anterior direction. After confirming the articular surface reduction by fluoroscopy, two cannulated lag screws were inserted from the anterior to posterior direction. In the case of the medial approach, a longitudinal incision was made from the tip of the medial malleolus. The great saphenous vein was protected. The medial malleolar fracture fragments were exposed. A periosteal detacher which was tightly attached to the surface of the tibia was used to perform blunt dissection until the posterior malleolus. Reduction was obtained by lifting and pulling the fracture fragments. Internal fixation was achieved by using the same method as that used for the posterolateral approach. After surgery, a gypsum plaster cast was used to immobilize the ankle joint. In addition, affected limb elevated exercise was routinely conducted to decrease the swelling. In addition, mannitol infusions (250 ml) were administered twice daily for 3 days and discontinued unless there was no obvious wound swelling. Four to six weeks postoperatively, patients were advised to perform functional exercises of the ankle joint.

### Clinical evaluation

Patients were followed up at 1 month, 3 month, 6 month and 12 months postoperatively. During the follow up period, anteroposterior and lateral X-ray radiographs or CT were obtained to assess the status of fracture healing and if there existed instrumentation failure and/or any recurrence of the disease. Rate of primary healing, healing time, incidence of talus necrosis, incidence of post-traumatic arthritis, functional outcomes, and any possible complications were recorded. Post-traumatic arthritis was considered to be achieved if there exists narrowing joint space, subchondral bone sclerosis, osteophyte formation and bone cystic degeneration [23]. The incidence of post-traumatic arthritis of the ankle was determined using imaging data by a senior author (ZG Zhang). Fracture healing was considered to be achieved when fracture line disappeared and bone trabecula crossing the fracture line.

Functional outcomes were evaluated using the Baird–Jackson scoring system [24]. The parameters observed included pain, ankle stability, walking ability, running ability, ankle movement range, and X-ray outcomes of the ankle. Functional outcomes were classified according to the Baird–Jackson scores as follows: 96–100 points, excellent; 91–95 points, good, 81–90, fair; and ≤ 80 points, poor.

### Statistical analysis

The SPSS 17.0 software (SPSS Inc., IL, USA) was used for data analysis. Quantitative data were expressed as means ± standard deviations (SD), and compared using the student *t*-test between two groups. Qualitative data are expressed as number or percentage and compared with the chi-squared test (χ^2^ test). A *P* value of less than 0.05 is considered as significant.

## Results

### Baseline data

A total of 69 patients, including 41 men and 28 women, with a mean age of 37.36 ± 15.23 years (range, 20–67 years) were included in this study. The patients were divided into an intraoperative ankle dislocation approach group (experimental group, *n* = 36) and a conventional approach group (control group, *n* = 33). The causes of ankle injury were found to be slip and fall in 38 patients and traffic accident in 31 patients. The fractures were associated with posterior dislocation of the talus in 37 patients (53.62%). All patients had concomitant distal tibiofibular separation. Sixteen patients had a history of failure of manual reduction. All fractures were acute closed injuries, and all posterior malleolar fractures were comminuted fractures, with the posterior malleolus fragments accounting for at least 25% of the articular surface. The ankle fractures were categorized using the Lauge–Hansen [3] classification including supination–external rotation type III fractures (39 patients) and pronation–external rotation type IV fractures (30 patients); and the Danis–Weber (AO/ASIF) classification [25] including type B fractures (27 patients) and type C fractures (42 patients). The time interval between injury and operation was 7.36 ± 2.72 days. The follow-up duration was 16.18 ± 11.03 months. No significant differences regarding sex, age, causes of injuries, incidence of concomitant posterior dislocation of talus, posterior ankle fracture block accounting for the area of the articular surface, Lauge-hansen classification, Danis-Weber classification, and duration from injury to surgery were observed between the experimental and control groups (all *P* > 0.05) (Table 1).

**Table 1:** The characteristics of the patients

	Experiment group (*n* = 36)	Control group (*n* = 33)	*P* values
Female/Male	22/14	19/14	0.821
Age (years)	36.36 ± 14.75	38.73 ± 12.18	0.945
BMI	23.92 ± 14.45	23.62 ± 13.25	0.921
Side: Left/Right	23/12	18/15	0.644
Cause: Strain/traffic injury (case)	20/16	18/15	0.876
Incidence of concomitant posterior dislocation of talus (%)	55.56% (20/36)	51.52% (17/33)	0.932
Posterior ankle fracture block accounts for the area of the articular surface (%)	42.16 ± 18.42	38.23 ± 20.73	0.522
Lauge-hansen system: supination-eversion type/ supination-external rotation type (case)	21/15	18/15	0.822
Danis-Weber system: B type/C type (case)	23/13	19/14	0.912
Time from injury to surgery (day)	7.62 ± 2.37	6.87 ± 3.18	0.774
Follow-up time (month)	17.69 ± 12.26	15.94 ± 10.66	0.681

### Comparison of clinical outcomes between experimental and control groups

**Fig. 9:**
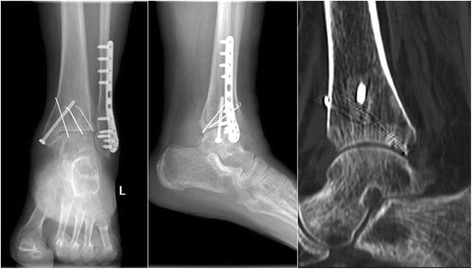
Postoperative X-ray and CT images 7 months after the surgery showed satisfactory fracture healing

**Fig. 10:**
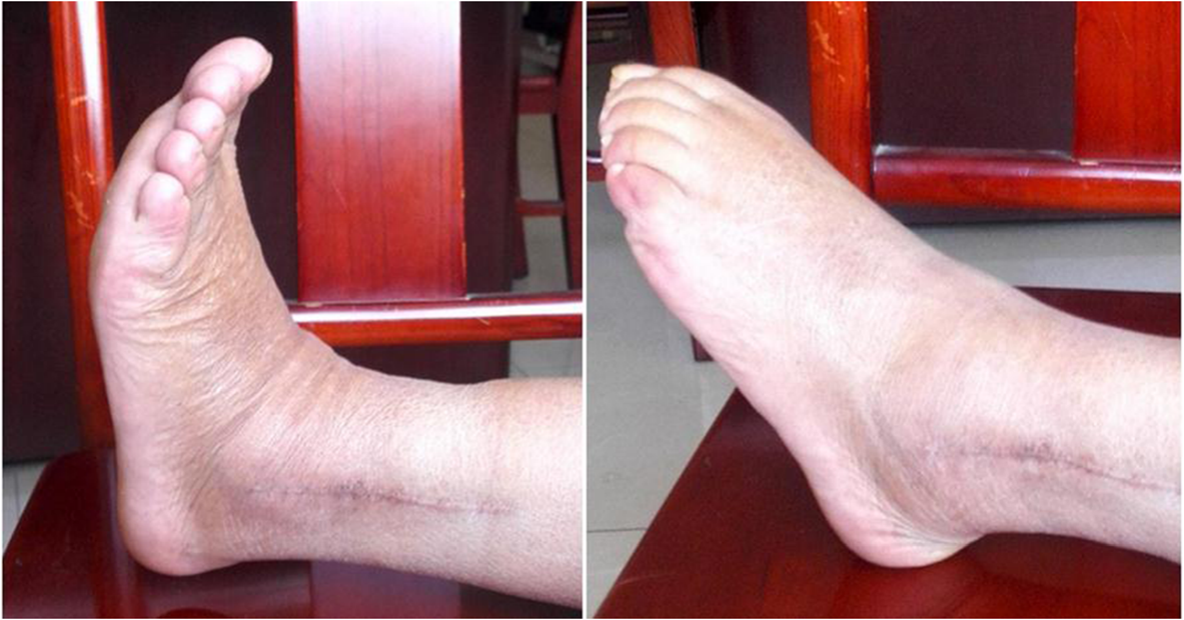
The pictures of ankle joint mobility showed the recovery of dorsal extension and plantar flexion

In the experimental group, primary wound healing was observed in 35 out of the 36 patients (97.22%), the secondary wound healing occurred in the remaining 1 patient. All patients in this group achieved the radiographic fracture healing. The mean healing time was 4.45 ± 1.63 months (range, 3 to 5 months). None of the patients developed necrosis of the talus. Functional outcomes in the experimental group were excellent in 27 patients, good in 6 patients, fair in 3 patients, and poor in 0 patients, respectively. Thus, the rate of excellent and good outcomes was 91.67% in the experimental group. Furthermore, in 33 of the 36 patients, ankle movements were sufficient to meet patients’ needs of the daily activities. Slight limitation of ankle movement was observed in 3 patients who were unable to squat. Thirty-six patients in the experimental group developed no post-traumatic arthritis. Five patients complained of ankle pain after excessive physical activities, but the pain was relieved after rest. No post-traumatic arthritis was seen on X-ray radiographs in these patients, and thus soft-tissue adhesion was considered to be the cause of the pain. In 4 of these 5 patients, the symptoms were relieved after 3 months of physiotherapy and functional exercises. The remaining patient continued to have pain after physiotherapy, but was less severe than before. A typical postoperative imaging results and ankle joint mobility image were shown in Figs. 9 and 10.

In the conventional treatment group, primary wound healing was achieved in all patients, none case occurred secondary wound healing. All patients in this group achieved the radiographic fracture healing. The healing time was 5.05 ± 1.94 months. Functional outcomes of patients in the control group were excellent in 16 patients, good in 8 patients, fair in 7 patients and poor in 2 patients, respectively. The rate of excellent and good outcomes was 72.73%. Three patients complained of ankle pain after excessive physical activities, but the pain was relieved after rest. No post-traumatic arthritis was seen on X-ray examinations in these patients, and the pain was attributed to soft-tissue adhesions. In 4 patients, the symptoms were considerably relieved after 3 months of physiotherapy and functional exercises; only one patient continued to have pain, but it was less severe than before. Eight patients had limitation of ankle movements and pain on weight bearing during walking; post-traumatic arthritis was confirmed in these patients on X-ray examination of the ankle at 9–22 months (13.12 ± 4.63 months) after the surgery. Two of these patients suffered severe pain that affected their daily lives; in these patients, the pain was reduced after ankle arthrodesis. In the remaining 6 patients, the pain was relieved by decreasing weight-bearing activities, but 3 of the patients required analgesic drugs.

No significant difference in the rate of primary healing was observed between the two groups (*P* > 0.05). The incidence of talus necrosis was 0% in both groups. The healing time also did not differ significantly between the two groups (*P* = 0.168). The incidence of post-traumatic arthritis was significantly lower in the experimental group than in the control group (0% vs. 24.24%, *P* = 0.006) (Table 2). Furthermore, the rate of excellent and good outcomes was significantly higher in the experimental group than in the control group (*P* = 0.038) (Table 3).

**Table 2:** The comparison of effects between experiment group and control group

	Experiment group (*n* = 36)	Control group (*n* = 33)	*P* values
Primary healing rate	97.22% (35/36)	100% (33/33)	0.993
Rate of talus necrosis	0% (0/36)	0% (0/33)	
Healing time (months)	4.45 ± 1.63	5.05 ± 1.94	0.168
Incidence of post-traumatic arthritis	0% (0/36)	24.24% (8/33)	0.006

**Table 3:** Functional outcomes in two groups

	Excellent	Good	Fair	Poor	Excellent and good rate	*P* values
Experiment group (*n* = 36)	27 (75%)	6 (16.67%)	3 (8.33%)	0 (0%)	33/36 (91.67%)	0.038
Control group (*n* = 33)	16 (48.48%)	8 (24.24%)	7 (21.21%)	2 (6.06%)	24/33 (72.73%)	

## Discussion

The trimalleolar fracture is the most complex ankle fracture. The treatment of posterior malleolus fracture is significant but less effective. Although the indications for the surgical treatment of posterior malleolar fractures are controversial [26], it is generally accepted that surgery is indicated for posterior malleolar fractures if it involves more than 25% of the articular surface or a more than 2-mm dislocation of the ankle joint, as observed on lateral X-ray examination of the ankle [27–30]. In the present study, all patients had unstable trimalleolar fractures involving comminuted posterior malleolar fracture, which is an absolute indication for surgery.

In the case of medial and lateral ankle fractures, the surgical approach is simple due to their superficial position. However, there is great controversy regarding the optimal approach for the fixation of posterior malleolar fracture. The conventional surgical approaches for posterior malleolar fractures include [3]: (1) In the case of the medial approach, subperiosteal dissection is performed to expose and reduce the posterior malleolar fracture via a medial incision. However, the posterior malleolus are usually exposed insufficiently, which can cause difficulty in achieving precise reduction and fixation. Therefore, this approach has currently been modified or replaced by other surgical approaches such as posteromedial or posterolateral approach. (2) The posterior approach can be used to fix the posterior malleolus alone. A lateral free skin flap or extension of the posterior incision is usually required to fix the lateral malleolus. However, there are two drawbacks that need to be noted. First, over-traction can easily cause neurovascular damage between the lateral malleolus and the Achilles tendon. In addition, it is hard to remove the internal fixation instruments after fracture healing due to local surgical scarring. (3) The posterolateral approach which involves a posterolateral incision is modified from conventional posterior approach and was first reported by Huber [19]. For this approach, an incision is made through the midline between the Achilles tendon and the fibula. Then, reduction and internal fixation of the posterior malleolar fracture can be done under direct vision. The lateral fracture can be reduced and fixed through the same incision. This approach decreases surgical injury and helps avoid disruption of the blood vessels and nerves within the tarsal tunnel. Furthermore, this modified approach can be used to simultaneously treat medial and lateral ankle fractures. Based on the pathological classification of posterior malleolar fractures proposed by Haraguchi et al. [18], the posterolateral approach is more suitable for type I fractures (posterolateral-oblique fractures), and the conventional medial approach or posteromedial approach is more advantageous for type II fractures (medial-extension fractures). Regarding the type III fractures (comminuted fractures, i.e., complicated posterior malleolar fractures), none of the above approaches can expose the articular surface sufficiently. Currently, little effort has been made to investigate the optimal treatment approaches for trimalleolar fractures involving posterior comminuted fractures and no definite criteria are available. In the present study, we introduced a novel intraoperative lateral ankle dislocation approach during surgical treatment for patients with unstable trimalleolar fractures involving posterior ankle comminuted fractures and compared its effects and safety with those with conventional approach. During follow-up, we found that the intraoperative ankle dislocation approach reduced the incidence of post-traumatic arthritis and improved ankle function but not compromised the rate of primary union, rate of talus necrosis, and union time. Moreover, it had the advantages of being a reproducible and simple operation.

Our technique has multiple advantages. Firstly, our technique can minimize the surgical damage. The syndesmotic tibiofibular ligaments, interosseous membranes and the posterior tibiofibular ligaments will be damaged when unstable trimalleolar fractures occurred caused by the rotating force. In addition, the function of the talocrural joint ligament, deltoid ligaments, and posterior tibiofibular ligament can also be impaired when the bony structures stabilizing the ankle were damaged. Consequently, the ankle becomes unstable. Therefore, the fracture itself caused a total injury of the stabilizing structures of the ankle. However, the intraoperative ankle dislocation approach will not damage the ankle-stabilizing structures and affect the anatomical structures surrounding the ankle, and thus can minimize the surgical damage. In addition, our technique allows reduction of the articular surface of the posterior malleolus under direct vision. The intraoperative dislocation of the ankle can sufficiently expose the articular surface of the posterior malleolus. Then, reduction and fixation of the comminuted fracture fragments of the posterior malleolus can be performed under direct vision. As stated in the surgical procedure section, EC gel was used for the adhesion of small fracture fragments connected with the articular surface. In our experience, the posterior malleolar fracture fragments attached to the posterior malleolar capsule is difficult to be completely dissected free, which will affect fracture reduction. Therefore, these fragments should be peeled from the distal ankle joint capsule to achieve fracture reduction and fixation. Although this might disrupt the blood supply, it ensured a smooth articular surface, which was beneficial to fracture union. Moreover, poor blood circulation did not cause nonunion or delayed union in our study. Thus, this type of free dissection is useful to improve prognosis in patients with trimalleolar ankle fractures. In the other surgical approaches, reduction of the articular surface could not be performed under direct vision. Furthermore, our technique did not increase the risk of the incidence of talus necrosis. Intraoperative lateral dislocation of the ankle did not affect the blood circulation of the talus. The blood supply of the talus mainly comes from the tarsal canal artery, the deltoid branch and posterior tubercle branch of the posterior tibial artery, the superior neck branches of the anterior tibial artery and tarsal sinus artery, and the perforating branch and root branch of the peroneal artery. These arteries are distributed around the non-articular surface of the talus, which forms an invisible arterial circle. Intraoperative ankle dislocation exposes only the lower part of the tibia, but not damages the arterial circle of the talus. Therefore, none of the talus necrosis occurred in our study.

Regarding the indications for intraoperative ankle dislocation, as stated above, the intraoperative ankle dislocation approach is most suitable for unstable trimalleolar fractures involving comminuted fractures of the posterior malleolus. Radiographic examinations showed that the lower part of the tibiofibular joint was completely separated and the posterior malleolar fracture was comminuted. In the case of relatively stable trimalleolar fractures, the intraoperative ankle dislocation approach will cause massive soft-tissue damage and is not a better choice. Therefore, there are limited indications for the intraoperative ankle dislocation approach. However, for complicated ankle fractures that require surgical treatment, this approach has obvious higher advantages over other approaches.

There are several limitations of this study. Firstly, the incidence rate of unstable trimalleolar fractures involving comminuted posterior malleolar fractures is not very high, which limits the wide application of the intraoperative ankle dislocation approach. Furthermore, the sample sizes in this study are relatively small, and the follow-up time was not very long. Therefore, whether or not there are long-term complications that were not observed in our study must be confirmed in future research. A prospective randomized controlled trial with a minimum follow up of 24 months is our future direction. Moreover, fibular fracture line will make a difference to the application of intraoperative ankle dislocation approach. The fibular fracture lines of our series are all above the distal tibiofibular syndesmosis, in other words, all patients had distal tibiofibular separation. Further clinical studies are required to determine whether this approach is also effective for other types of trimalleolar fractures whose fibular fracture lines are below the tibiofibular syndesmosis (without distal tibiofibular separation).

## Conclusions

The findings suggest that the intraoperative ankle dislocation approach appears to be a promising surgical option for unstable trimalleolar fractures involving posterior ankle comminuted fracture because it can provide better functional outcomes and lower incidence of post-traumatic arthritis while not compromising primary healing and healing time. Large-scale prospective randomized controlled trials and multi-institutional studies are required to confirm and modify our findings in the future.
